# Water Quality Prediction Based on Multi-Task Learning

**DOI:** 10.3390/ijerph19159699

**Published:** 2022-08-06

**Authors:** Huan Wu, Shuiping Cheng, Kunlun Xin, Nian Ma, Jie Chen, Liang Tao, Min Gao

**Affiliations:** 1 College of Environmental Science and Engineering, Tongji University, Shanghai 200092, China; 2 T.Y.Lin International Engineering Consulting (China) Co., Ltd., Chongqing 401121, China; 3 Faculty of Natural Sciences, University of the Western Cape, Cape Town 7535, South Africa; 4 College of Environment and Ecology, Chongqing University, Chongqing 400030, China; 5 School of Big Data and Software Engineering, Chongqing University, Chongqing 401331, China

**Keywords:** multi-task learning, water quality prediction, multiple indicator prediction

## Abstract

Water pollution seriously endangers people’s lives and restricts the sustainable development of the economy. Water quality prediction is essential for early warning and prevention of water pollution. However, the nonlinear characteristics of water quality data make it challenging to accurately predicted by traditional methods. Recently, the methods based on deep learning can better deal with nonlinear characteristics, which improves the prediction performance. Still, they rarely consider the relationship between multiple prediction indicators of water quality. The relationship between multiple indicators is crucial for the prediction because they can provide more associated auxiliary information. To this end, we propose a prediction method based on exploring the correlation of water quality multi-indicator prediction tasks in this paper. We explore four sharing structures for the multi-indicator prediction to train the deep neural network models for constructing the highly complex nonlinear characteristics of water quality data. Experiments on the datasets of more than 120 water quality monitoring sites in China show that the proposed models outperform the state-of-the-art baselines.

## 1. Introduction

The excessive exploitation and utilization of water resources have caused a series of problems, such as deterioration of water quality, damage to water functional areas, and degradation of river ecosystem structures, which seriously endanger the social and economic development and the safety of people. Water quality prediction is essential for water pollution prevention and treatment, which can help fully understand the dynamic trend of the surface water ecological environment and warn of possible pollution incidents.

However, it is difficult to predict water quality because of the nonlinear characteristics of water-related data [1]. Traditional statistical analysis methods lack nonlinear approximation and self-learning abilities and cannot fully consider the complex impact of various environmental factors. With the rapid development of machine learning technology, scholars have begun to explore water quality prediction based on machine learning. They have achieved better water quality prediction performance by establishing nonlinear learning cognitive models from historical data, summarizing and discovering knowledge, and predicting system behavior. Olyaie et al. (2017) applied linear genetic programming and a support vector machine (SVM) to predict dissolved oxygen (DO) in the Delaware River in Trenton, USA [2]. Li et al. (2017) proposed a method that combines ensemble empirical mode decomposition (EEMD) [3,4,5] and least-squares SVR (support vector regression) to predict DO concentration [6]. Leong et al. (2021) applied SVM [7,8,9] and least squares support vector models to the Perak River in Malaysia [10]. The performance of these methods depends not only on the models but also on the features selected for training.

More and more researchers have recently applied deep learning methods to water quality prediction because deep learning (DL) [11,12] can efficiently train and abstract multi-level features of multi-dimensional training data. Banejad et al. (2011) applied a basic neural network to predict biochemical oxygen demand (BOD) and DO of the Morad River in Iran [13]. They verified that the deep learning technology can reliably, efficiently, and accurately extract the nonlinear characteristics of water quality data. Subsequently, Heddam et al. (2014, 2016) and Liu et al. (2020) successively proposed models based on GRNN (generalized regression neural network) and MLP (multilayer perceptron), which were applied to different rivers in the United States and lakes in China [14,15,16]. Zhou et al. (2019) proposed a deep cascade forest (DCF) that uses several random forests based on ensemble learning, performing well on large and even small-scale data [17]. Wang et al. (2019) proposed a hybrid CNN-LSTM (convolutional neural network- long short-term memory) deep learning algorithm for a dynamic chemical oxygen demand (COD) prediction model of urban sewage [18]. Zou et al. (2020) proposed a water quality prediction method based on the Bi-LSTM (Bidirectional LSTM) model with multiple time scales [19]. Niu et al. (2021) also developed a pixel-based deep neural network regression model and a patch-based deep neural network regression model, to estimate seven optically inactive water quality parameters [20]. Yang et al. (2021) proposed a mixed model named CNN-LSTM with Attention (CLA), combining CNN, LSTM, and Attention mechanisms to predict water quality [21]. Guo et al. (2022) use progressively decreasing deep neural network and multimodal deep learning (MDL) models without well-handled input features, to estimate long-term water indicators and explore the contribution of each feature by quantifying [22].

However, these models are constructed to optimize a single prediction indicator such as the potential of hydrogen (pH), dissolved oxygen (DO), chemical oxygen demand-Mn (COD_Mn_), and Ammonia Nitrogen (NH_3_-N, NHN for short), etc., which cannot guarantee the high efficiency and accuracy of the models in predicting other water quality indicators. The correlation between multiple prediction indicators can provide more correlation auxiliary information, which helps improve the prediction performance. To this end, we propose a water quality prediction model based on multi-task learning by learning the highly complex nonlinear characteristics of time series data and exploring the correlation of multi-indicator prediction.

The main contributions of this paper are as follows:

(1) We propose a multi-indicator prediction model of surface water quality based on deep learning, which excavates the highly complex nonlinear characteristics of surface water ecological environment water quality data and explores the correlation of multiple water quality prediction indicators.

(2) We propose four water quality prediction frameworks, named hard parameter sharing structure (Multi-Task-Hard), soft parameter sharing structure (Multi-Task-Soft), gated parameter sharing structure (Multi-Task-Gate), and gated hidden parameters sharing structure (Multi-Task-GH), based on different multi-task learning structures and combine the frameworks with various mainstream deep learning models to form different water quality prediction models.

(3) We conducted experiments to predict four water quality indicators, including pH, DO, COD_Mn_, and NH_3_-N, on real data from more than 120 water quality monitoring sites in seven river systems and lakes in China. The experimental results demonstrate that the proposed water quality multi-task learning prediction framework outperforms the state-of-the-art single-indicator prediction models.

## 2. Methodology

The existing deep learning-based water quality prediction models rarely consider the relationship between multiple indicators of water quality. The relationship between multiple indicators is crucial for the prediction because they can provide more associated auxiliary information. To this end, we propose a prediction method based on exploring the correlation of water quality multi-indicator prediction tasks in this section. We first define the water quality prediction and explore four sharing structures for the multi-indictor prediction to train the deep neural network models for constructing the highly complex nonlinear characteristics of water quality data.

### 2.1. Definition of Water Quality Prediction

Following previous work [19,23,24], we choose four water quality indicators, including pH, DO, COD_Mn_, and NH_3_-N, as our prediction targets. Compared with other indicators, these indicators can predict that six water quality levels perform significantly better, reflecting the water quality better [23].

The water quality prediction is a time series prediction. We give the mathematical definitions for single-task prediction and multi-task prediction.

Single-task water quality prediction:  X→Y. Given water quality prediction indicators at known past times $(x_{1},\ldots ,x_{i})\in X$, analyze the change patterns and predict the water quality indicator at the future time interval [11], denoted as $y_{i+1}\in Y$.

Multi-task water quality prediction: $(X_{1},\ldots ,X_{N})\to \left(Y_{1},\ldots ,Y_{N}\right)$. Given *N* water quality prediction indicators of the past *i* times $\left\{(x_{11},\ldots ,x_{i1}),\ldots ,(x_{1N},\ldots ,x_{iN})\}\in (X_{1},\ldots ,X_{N}\right)$, analyze the change patterns of N indicators at the same time, and predict multiple water quality indicators at the future time interval, denoted as $\left(y_{1},\ldots ,y_{N}\right)\in \left(Y_{1},\ldots ,Y_{N}\right)$.

For the four common water quality prediction indicators, pH, CODMn, DO, and NH3-N, the multi-task water quality prediction task can be defined as, given the numerical changes of pH, DO, COD_Mn_, NH_3_-N at the past *i* time intervals $\left\{(x_{pH1},\ldots ,x_{pHi}),(x_{DO1},\ldots ,x_{DOi}),(x_{COD1},\ldots ,x_{CODi}),(x_{NHN1},\ldots ,x_{NHNi})\}\in (X_{pH},X_{CO},X_{COD},X_{NHN}\right)$, analyze the change patterns and predict the corresponding water quality indicators at the future time interval, denoted as $\left(y_{pH},y_{DO},y_{COD},y_{NHN}\right)\in \left(Y_{pH},Y_{DO},Y_{COD},Y_{NHN}\right)$.

### 2.2. Architecture of Water Quality Prediction Model Based on Multi-Task Learning

**Figure 1:**
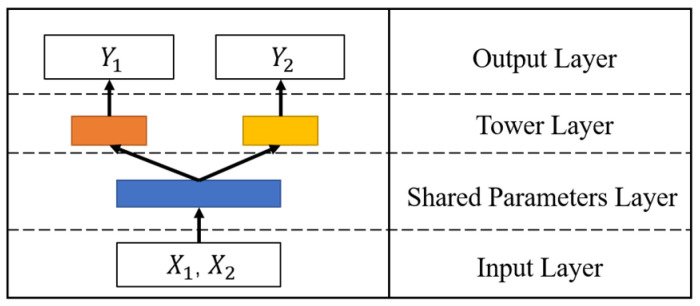
The basic framework of multi-task learning. The blue part represents the shared parameter layer, and the orange and yellow parts represent the models for different tasks forming the tower layer.

Frameworks for multi-task learning are often based on sharing the same bottom structure [25,26,27]. The model of multiple tasks can be transformed into a basic bottom model and multiple separate models. For single-task learning, the input and output of each task correspond to a separate model, and new models need to be built for new tasks, although the structure of the models is sometimes the same. For multi-task learning, the common structure of the model is unified into a basic model. Then, several separate models are introduced to realize the learning of multiple different tasks. Figure 1 is a basic framework for multi-task learning, in which the blue part represents the shared parameter layer, and the orange and yellow parts represent models for different tasks forming the tower layer. This framework structure saves the parameter space of multiple water quality prediction models and reduces the risk of over-fitting. We propose a multi-task learning framework for water quality prediction based on different structures [28,29]. The framework can be developed into four forms: hard parameter sharing structure (Multi-Task-Hard), soft parameter sharing structure (Multi-Task-Soft), gated parameter sharing structure (Multi-Task-Gate), and gated hidden parameters sharing structure (Multi-Task-GH). The differences between the four structures are described in detail in Section 2.3.

### 2.3. Multi-Task Learning Structures

#### 2.3.1. Hard Parameter Sharing Structure of Multi-Indicator Water Quality Prediction (Multi-Task-Hard)

**Figure 2:**
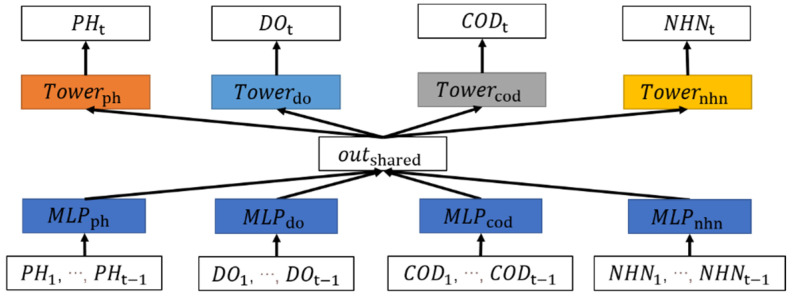
Hard parameter sharing structure of multi-indicator water quality prediction. The dark blue part represents the shared parameter layer, and the orange and yellow parts represent the models for different tasks forming the tower layer.

The hard parameter sharing structure is the basic structure of the shared bottom structure in multi-task learning. As shown in Figure 2, it is mainly divided into four parts.

The first part is the input layer $\left(X_{1},\ldots ,X_{N}\right)$, which contains the time sequence information of each water quality indicator at the past time intervals.

The second part is the shared parameter layer, which is designed as a fully connected layer. This part takes the information transmitted by the input layer and extracts a shared implicit vector $out_{shared}$.

The third part is the tower layer, which is carefully designed for a task and will output the prediction results required by the corresponding task, which reflects the flexibility of the multi-task learning framework. The output of the second layer will be transmitted to the tower layer for different tasks simultaneously. Because of the differences between the indicators, it is necessary to design specific models for different water quality indicators in this layer. To put it simply, one task corresponds to one tower.

The fourth part is the output layer, which contains the outputs: $Y_{pH},Y_{DO},Y_{COD},\;Y_{NHN}$ as the prediction.

The algorithm is shown in Algorithm 1, and the MLP is selected for processing in the shared layer.
**Algorithm 1:** Multi-indicator water quality prediction based on hard parameter sharing multi-task learningInput: water quality prediction indicators at the past time intervals $\left(X_{1},\ldots ,X_{N}\right)$ $1:\;out_{shared}\leftarrow \mathrm{MLP}([X_{1},\ldots ,X_{N}])$ $2:\;(Y_{1},\ldots ,Y_{N})\leftarrow {\mathrm{Tower}}_{1,\ldots ,N}\left(out_{shared}\right)$ Output: water quality indicators at the future time intervals $\left(Y_{1},\ldots ,Y_{N}\right)$

We introduce the specific structure of the hard parameter sharing structure with pH, DO, COD_Mn_, and NH_3_-N as the prediction target. As shown in Figure 2, all indicators from the input layer to the shared parameter layer have the same structure. Take the pH value part as an example. We input data $\left({pH}_{1},\ldots ,{pH}_{t-1}\right)$ in the input layer, which will be transmitted to the shared parameter layer and converted into the output vector by the fully connected neural network. Similarly, the inputs of DO, COD_Mn_, and NH_3_-N will also be converted to output vectors $out_{DO},out_{COD},out_{NHN}$ accordingly. The equation is as Equation (1). All input data will be dealt with by MLP and ReLU (rectified linear unit).

(1) $$out_{pH}=\mathrm{ReLU}(\mathrm{MLP}({pH}_{1},\ldots {pH}_{t-1}))\;out_{DO}=\mathrm{ReLU}(\mathrm{MLP}({DO}_{1},\ldots {DO}_{t-1}))\;out_{COD}=\mathrm{ReLU}(\mathrm{MLP}({COD\mathrm{Mn}}_{1},\ldots {COD\mathrm{Mn}}_{t-1}))\;out_{NHN}=\mathrm{ReLU}(\mathrm{MLP}(NHN_{1},\ldots NHN_{t-1}))$$

where ReLU is a nonlinear function used to add nonlinearity to the model. Compared with sigmoid, ReLU can effectively alleviate the problems of gradient disappearance and gradient explosion in deep neural networks. The formula of ReLU is shown in Equation (2):

(2) $$\mathrm{ReLU}\left(x\right)=\{\begin{array}{c} x,\;\;if\;x>0\\ 0,\;\;if\;x\leq 0 \end{array}$$

We use the multilayer perceptron (MLP) to extract the deeply hidden features of the water quality time series. MLP can simply and efficiently represent the global features of time series, which helps the subsequent tower layer extract the deep local features for different water quality indicators. The formulation of MLP is shown in Equations (3) and (4), where *x* denotes the input, W denotes the weight matrix $w_{i}$, *b* denotes the bias term, and *y* denotes the final output.

(3) $$z=\overset{n}{\underset{i=1}{\sum }}w_{i}x_{i}+b$$

(4) y=ReLU(z)

The MLP model consists of three parts: input layer, hidden layer, and output layer. The number of hidden layers in the MLP can be adjusted as a hyperparameter. The number of neurons in the output layer is the number of the water quality prediction indicators. We train the MLP model with the BP (Back Propagation) algorithm, whose loss propagates back from the top layer to the bottom layer.

The last layer of the network is the output layer, and the loss function is defined as Equation (5), where $L_{n}$ represents all neurons of the layer, $y_{n}^{\left(j\right)}$ represents the output of the *j*-th neuron, t denotes the predicted value corresponding to $\left({\hat{pH}}_{t},{\hat{DO}}_{t},{\hat{COD\mathrm{Mn}}}_{t},\hat{{NHN}_{t}}\right)$, and *y* denotes the real value corresponding to $\left({pH}_{t},{DO}_{t},{COD\mathrm{Mn}}_{t},{NHN}_{t}\right)$.

(5) $$Loss=\frac{1}{2}\sum_{j\in L_{n}}{\left(t^{\left(j\right)}-y_{n}^{\left(j\right)}\right)}^{2}$$

The variables *w* and *b* are obtained by gradient descent to minimize the loss function, we show Equations (6)–(8) below to show the calculation of *w* and *b*’s gradient:

(6) $$\frac{\partial Loss}{\partial w_{l}^{\left(ji\right)}}=\frac{\partial Loss}{\partial y_{l}^{\left(j\right)}}\frac{\partial y_{l}^{\left(j\right)}}{\partial w_{l}^{\left(ji\right)}}=\frac{\partial Loss}{\partial y_{l}^{\left(j\right)}}\frac{\partial y_{l}^{\left(j\right)}}{\partial z_{l}^{\left(j\right)}}\frac{\partial z_{l}^{\left(j\right)}}{\partial w_{l}^{\left(ji\right)}}=\delta_{l}^{\left(j\right)}y_{l-1}^{\left(i\right)}$$

(7) $$\frac{\partial Loss}{\partial b_{l}^{\left(j\right)}}=\frac{\partial Loss}{\partial y_{l}^{\left(j\right)}}\frac{\partial y_{l}^{\left(j\right)}}{\partial b_{l}^{\left(j\right)}}=\frac{\partial Loss}{\partial y_{l}^{\left(j\right)}}\frac{\partial y_{l}^{\left(j\right)}}{\partial z_{l}^{\left(j\right)}}\frac{\partial z_{l}^{\left(j\right)}}{\partial b_{l}^{\left(j\right)}}=\delta_{l}^{\left(j\right)}$$

(8) $$\delta_{l}^{\left(j\right)}=\frac{\partial Loss}{\partial y_{l}^{\left(j\right)}}f^{\prime }(z_{l}^{\left(j\right)})=f^{\prime }(z_{l}^{\left(j\right)})\sum_{k\in L_{l+1}}\delta_{l+1}^{k}w_{l+1}^{\left(kj\right)}$$

(9) $$W_{l}\leftarrow W_{l}-\eta \frac{\partial Loss}{\partial W_{l}}=W_{l}-\eta \delta_{l}y_{l-1}^{T}$$

(10) $$b_{l}\leftarrow b_{l}-\eta \frac{\partial Loss}{\partial b}=b_{l}-\eta \;\delta_{l}$$

(11) $$out_{shared}=concat\left(out_{pH},out_{DO},out_{COD},out_{NHN}\right)$$

(12) $${\hat{pH}}_{t}=\mathrm{ReLU}({\mathrm{MLP}}_{pH}(out_{shared})),\;{\hat{DO}}_{t}=\mathrm{ReLU}({\mathrm{MLP}}_{DO}(out_{shared})),\;{\hat{CODMn}}_{t}=\mathrm{ReLU}({\mathrm{MLP}}_{COD}(out_{shared})),\;\hat{NHN_{t}}=\mathrm{ReLU}({\mathrm{MLP}}_{NHN}(out_{shared})).$$

(13) $$Loss=\sqrt{\frac{1}{N}\overset{N}{\underset{i=1}{\sum }}{\left(pH_{ti}-{\hat{pH}}_{ti}\right)}^{2}+{\left(DO_{ti}-{\hat{DO}}_{ti}\right)}^{2}+{\left(COD_{ti}-{\hat{COD}}_{ti}\right)}^{2}+{\left(NHN_{ti}-{\hat{NHN}}_{ti}\right)}^{2}}$$

The parameters update formulas of each layer are expressed in matrix forms, as shown in Equations (9) and (10):

The well-trained model consists of updated *w* and *b* finally, and the output of Equation (1) can be obtained. Concatenating the four output vectors of Equation (1) to obtain, as shown in Equation (11), we can obtain the $out_{shared}.$

$out_{shared}$ is the input of different tower layers (pH, DO, COD_Mn_, and NH_3_-N correspond to different towers) to generate corresponding prediction. Due to the different prediction targets, the tower layer structure can be different. Although the input $out_{shared}$ is the same for all towers, the output of each tower layer is different. The formulas are shown in Equation (12):

Finally, the Root Mean Square Error (RMSE) between the predicted values $({\hat{pH}}_{t},{\hat{DO}}_{t},{\hat{COD\mathrm{Mn}}}_{t},\hat{{NHN}_{t}})\;$ and the real values $\left({pH}_{t},{DO}_{t},{COD\mathrm{Mn}}_{t},{NHN}_{t}\right)$ is calculated as the loss (see Equation (13)), where N is the number of samples. The loss is backpropagated to update the model parameters until the model converges.

All tasks share a shared parameter layer in the hard parameter sharing structure, and different tower layers are built for different tasks. Such structure reduces the complexity of the model structure and parameters. It ensures the model’s flexibility since the model is required to learn a general implicit embedding in the sharing layer to make each task perform better, thus reducing the risk of overfitting.

#### 2.3.2. Soft Parameter Sharing Structure of Multi-Indicator Water Quality Prediction (Multi-Task-Soft)

The shared parameter layer of Multi-Task-Hard cannot reflect the relationship between different tasks well and cannot guarantee the stable performance of the model. Therefore, we propose a soft parameter sharing structure-based multi-indicator water quality prediction (Multi-Task-Soft), which is based on Multi-Task-Hard. In the Multi-Task-Soft, data will be input to modules of different tasks to extract different features. Different tasks jointly maintain an implicit vector to learn the correlation between different indicators.

**Figure 3:**
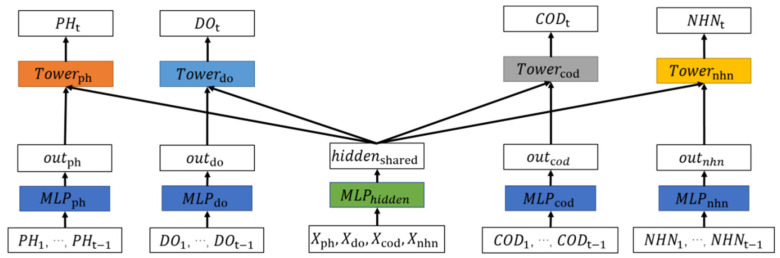
Soft parameter sharing structure of multi-indicator water quality prediction.

The architecture of Multi-Task-Soft is similar to that of the Multi-Task-Hard, as shown in Figure 3, which is also composed of four parts. Their main difference is the design of the shared parameter layer. Different from the single parameter sharing layer of Multi-Task-Hard, Multi-Task-Soft inputs the data to modules of different tasks to obtain different outputs. The structure also maintains an implicit vector to learn the correlation between different indicators. The implicit vector is merged with the outputs corresponding to the underlying structures of each task, and the merged results are input to the tower layer. Finally, each tower model will output the prediction results required by the corresponding task. The model process is shown in Algorithm 2.
**Algorithm 2:** Multi-indicator water quality prediction based on soft parameter sharing multi-task learningInput: water quality prediction indicators at the past time intervals $\left(X_{1},\ldots ,X_{N}\right)$ $1:\;{hidden}_{shared}\leftarrow {\mathrm{MLP}}_{hidden}([X_{1},\ldots ,X_{N}])$ $2:\;({\mathrm{out}}_{1},\ldots ,{\mathrm{out}}_{n})\leftarrow {\mathrm{MLP}}_{1,\ldots ,n}\left(X_{1},\ldots ,X_{N}\right)$ $3:\;(Y_{1},\ldots ,Y_{n})\leftarrow {\mathrm{Tow}}_{1,\ldots ,n}\left(\left[{\mathrm{out}}_{1,\ldots ,n},{\;\mathrm{out}}_{shared}\right]\right)$ Output: water quality indicators at the future time intervals $\left(Y_{1},\ldots ,Y_{N}\right)$

Input $(pH_{1},\ldots ,\;pH_{t-1})\in X_{pH},\;(DO_{1},\ldots ,DO_{t-1})\in X_{DO},\;\left(COD_{Mn_{1}},\ldots ,COD_{Mn_{t-1}}\right)\in X_{COD}$, and $\left(NHN_{1},\ldots ,HNH_{t-1}\right)\in X_{NHN}$ to the model. The data is passed through the fully connected neural network (as shown in Equation (1)) to obtain the output vectors ($out_{pH},out_{DO},out_{COD},out_{NHN}$), respectively.

Meanwhile, ($X_{pH}$, $X_{DO}$, $X_{COD}$, $X_{NHN}$) is also used as the input of another fully connected neural network to obtain the output vector $hidden_{shared}$, as shown in Equation (14):

(14) $$hidden_{shared}=\mathrm{ReLU}(\mathrm{MLP}(X_{pH},\;X_{DO},\;X_{COD},\;X_{NHN})).$$

Concatenate output vectors and $hidden_{shared}$ to obtain corresponding vectors $v_{pH},v_{DO},v_{COD},v_{NHN}$.

(15) $$v_{pH}=concat\left(out_{pH},hidden_{shared}\right),\;v_{DO}=concat\left(out_{DO},hidden_{shared}\right),\;v_{COD}=concat\left(out_{COD},hidden_{shared}\right),\;v_{NHN}=concat\left(out_{NHN},hidden_{shared}\right).$$

Then, we input the vectors to the corresponding tower layer. Similar to Multi-Task-Hard, pH, DO, COD_Mn_, and NH_3_-N correspond to different towers, and the tower layer can be any neural network structure model. For different prediction indicators, the tower layer structure is different, which makes the corresponding output different. Taking MLP as an example, the predictions are shown as Equation (16):

(16) $$\hat{{pH}_{t}}=\mathrm{ReLU}({\mathrm{MLP}}_{pH}(v_{pH})),\;\hat{{DO}_{t}}=\mathrm{ReLU}({\mathrm{MLP}}_{DO}(v_{DO})),\;\hat{{COD}_{t}}=\mathrm{ReLU}({\mathrm{MLP}}_{COD}(v_{COD})),\;\hat{{NHN}_{t}}=\mathrm{ReLU}({\mathrm{MLP}}_{NHN}(v_{NHN})).$$

Finally, the RMSE between the predicted and real values is calculated as the loss, and the model parameters are updated by the backpropagation method until the model converges.

In this structure, the association between different indicators is obtained by learning an implicit public vector, and each task has its unique learning module. Finally, the individual learning and joint learning results are merged to achieve better prediction results.

#### 2.3.3. Gating Parameter Sharing Structure of Multi-Indicator Water Quality Prediction (Multi-Task-Gate)

**Figure 4:**
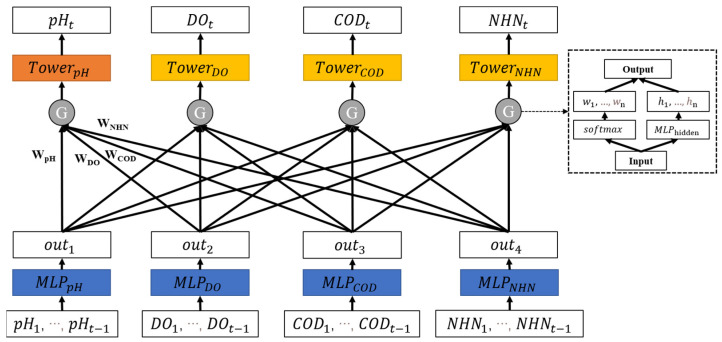
Gating parameter sharing structure of multi-indicator water quality prediction.

To better learn the relative weight of different indicators for the task, we further add the gating module in the parameter sharing layer. As shown in Figure 4, the input is processed by different modules to obtain different implicit features. The implicit features obtain the weight of the current task through SoftMax. According to the weight, different implicit vectors are weighted and summed to obtain the tower layer input of each task. Finally, each tower model outputs the prediction results of the corresponding task. The model process is shown as Algorithm 3.

The design of the shared parameter layer is similar to Multi-Task-Hard, and the data $\left(pH_{1},\ldots ,pH_{t-1})\in X_{pH},\;(DO_{1},\ldots ,DO_{t-1})\in X_{DO},\;(COD_{Mn_{1}},\ldots ,\;COD_{Mn_{t-1}}\right)$ $\in X_{COD},\left(NHN_{1},\ldots ,HNH_{t-1}\right)\in X_{NHN}$ is input separately to the fully connected neural network of the shared parameter layer to obtain the output vectors $out_{pH},out_{DO},out_{COD},out_{NHN}$, respectively. It is shown as Equation (1).

Unlike Multi-Task-Hard, the module calculates the importance of different output vectors to predict the pH instead of concatenating them and feeding them to the tower layer. Taking the prediction of pH as an example, we obtain the relative weights of different indicators in the prediction of pH through softmax. Softmax can map relative weights $\;(w_{pH},w_{DO},w_{COD},w_{NHN})$ from 0 to 1. The relative weights show the corresponding results of different indicators, as shown in Equation (17):

(17) $$\left(w_{pH},w_{DO},w_{COD},w_{NHN}\right)=\mathrm{softmax}\;(MLP_{ph}\left(out_{pH},out_{DO},out_{COD},out_{NHN}\right).$$

Meanwhile, the output vectors ($out_{pH},out_{DO},out_{COD},out_{NHN})$ are mapped through an MLP to $\left(hidden_{pH},hidden_{DO},hidden_{COD},hidden_{NHN}\right)$, as shown in Equation (18):

(18) $$hidden_{pH},hidden_{DO},hidden_{COD},hidden_{NHN}={\mathrm{MLP}}_{\mathrm{hidden}}\left(out_{pH},out_{DO},out_{COD},out_{NHN}\right).$$

The vector input of the tower layer is obtained by weighted fusion, as shown in Equation (19):

(19) $$v_{pH}=W_{pH}\times hidden_{pH}+W_{COD}\times hidden_{COD}+W_{DO}\times hidden_{DO}+W_{NHN}\times hidden_{NHN}.$$

Similarly, the tower layers of DO, COD_Mn_, and NH_3_-N also obtain the corresponding inputs, and the tower layers are designed as MLP. For different prediction indicators, the tower layer structure and output can be different. Taking MLP as an example, the formula is shown as Equation (16) in Section 2.3.2.

Finally, the RMSE between the predicted value and the real value is calculated as the loss, and the model parameters are updated by the backpropagation method until the model converges.

The gating parameter sharing structure does not learn the implicit vectors to extract the connection between tasks but learns the importance and connection of different indicators relative to a single task through the gating mechanism, which improves prediction performance.
**Algorithm 3:** Multi-task Learning of Gating Parameter Sharing Structure for Multi-indicator Water Quality PredictionInput: water quality prediction indicators at the past time intervals $\left(X_{1},\ldots ,X_{N}\right)$ $1:\;(hidden_{1},\ldots ,hidden_{n})\leftarrow {\mathrm{MLP}}_{1,\ldots ,n}([X_{1},\ldots ,X_{N}])$ $2:\;(w_{i1},\ldots ,w_{in})\in W_{i}\leftarrow \mathrm{Softmax}({\mathrm{MLP}}_{shared}^{i}(hidden_{1},\ldots ,hidden_{n}))$ $3:\;(Y_{1},\ldots ,Y_{n})\leftarrow {\mathrm{Tow}}_{i}\left(\left[\sum_{i}^{n}W_{i}\times hidden_{i}\right]\right)$ Output: water quality indicators at the future time intervals $\left(Y_{1},\ldots ,Y_{N}\right)$

#### 2.3.4. Gated Hidden Parameter Sharing Structure of Multi-Indicator Water Quality Prediction (Multi-Task-GH)

**Figure 5:**
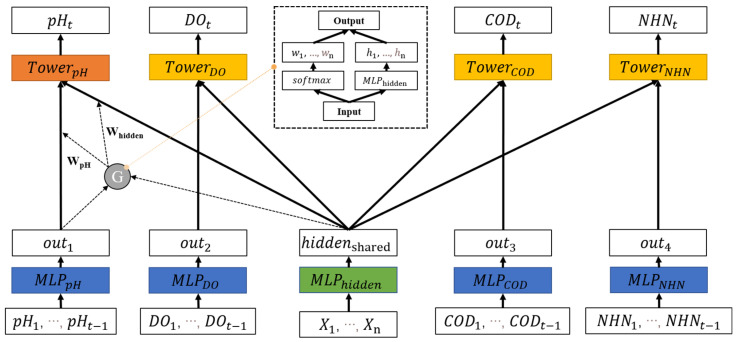
Gated hidden parameter sharing structure of multi-indicator water quality prediction.

**Figure 6:**
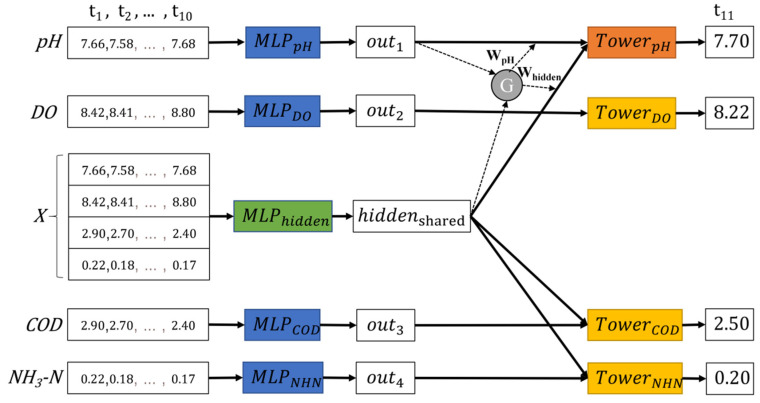
An example of the kind of time series for each indicator.

This section proposes a multi-task learning structure, which combines the advantages of the soft parameter sharing structure and the gated parameter sharing structure. As shown in Figure 5, the structure of the gated hidden parameter sharing structure (Multi-Task-GH) is similar to the Multi-Task-Gate, except that there is a model for learning an intermediate hidden vector in the parameter sharing layer. This intermediate implicit vector is similar to the Multi-Task-Soft design, which is combined with all other implicit vectors. The output results will be input to the tower layer through the gating mechanism. Finally, each tower model outputs the prediction results of the corresponding task. The model algorithm process is shown in Algorithm 4. Figure 6 shows an example of the kind of time series for each indicator.
**Algorithm 4:** Gated Hidden Parameter Sharing Structure Multi-Task Learning for Multi-indicator Water Quality PredictionInput: water quality prediction indicators at the past time intervals $\left(X_{1},\ldots ,X_{N}\right)$ $1:\;hidden_{shared}\leftarrow {\mathrm{MLP}}_{hidden}([X_{1},\ldots ,X_{N}])$ $2:\;(out_{1},\ldots ,out_{n})\leftarrow {\mathrm{MLP}}_{1,\ldots ,n}\left(X_{1},\ldots ,X_{N}\right)$ $3:\;(w_{i1},\ldots ,w_{in})\in W_{i}\leftarrow \mathrm{Softmax}({\mathrm{MLP}}_{shared}^{i}([out_{i},hidden_{shared}]))$ $4:\;(Y_{1},\ldots ,Y_{n})\leftarrow {\mathrm{Tow}}_{i}\left(\left[\sum_{i}^{n}W_{i}*\left[out_{i},hidden_{shared}\right]\right]\right)$ Output: water quality indicators at the future time intervals $\left(Y_{1},\ldots ,Y_{N}\right)$

The design of the shared parameter layer is similar to Multi-Task-Soft, and data is input separately to the input layer: $(pH_{1},\ldots ,pH_{t-1})\in X_{pH},\;(DO_{1},\ldots ,DO_{t-1})\in X_{DO},\;(COD_{Mn_{1}},\ldots ,\;COD_{Mn_{t-1}})\in X_{COD}$, and $\left(NHN_{1},\ldots ,HNH_{t-1}\right)\in X_{NHN}$. The data is passed to MLP of the shared parameter layer to obtain the output vectors, respectively. It is shown in Equation (1).

We then input $(X_{pH}$, $X_{DO}$, $X_{COD}$, $X_{NHN})$ to another implicit vector MLP to obtain the output vector $hidden_{shared}$. As shown in Equation (20):

(20) $$hidden_{shared}=\mathrm{ReLU}\left(\mathrm{MLP}\left(X_{pH},\;X_{DO},\;X_{COD},\;X_{NHN}\right)\right)$$

Unlike Multi-Task-Soft, the module calculates the importance of different vectors $out_{pH}$, $out_{DO}$, $out_{cod}$ and $out_{nhn}$ for the prediction target together with $\;hidden_{shared}$, respectively. The relative weight of the predicted target is obtained through Softmax, as shown in Equation (21):

(21) $$\left(w_{pH},w_{hidden}\right)=\mathrm{Softmax}\;({\mathrm{MLP}}_{pH}\left(out_{pH},hidden_{shared}\right)),\;\left(w_{DO},w_{hidden}\right)=\mathrm{Softmax}\;({\mathrm{MLP}}_{DO}\left(out_{DO},hidden_{shared}\right)),\;\left(w_{COD},w_{hidden}\right)=\mathrm{Softmax}\;({\mathrm{MLP}}_{COD}\left(out_{COD},hidden_{shared}\right)),\;\left(w_{NHN},w_{hidden}\right)=\mathrm{Softmax}\;({\mathrm{MLP}}_{NHN}\left(out_{NHN},hidden_{shared}\right)).$$

Meanwhile, the output vectors are mapped through a fully connected neural network to $\left(hidden_{pH},hidden_{DO},hidden_{COD},hidden_{NHN}\right)$:

(22) $$hidden_{pH}=\mathrm{ReLU}\left({\mathrm{MLP}}_{hidden}\left(out_{pH},hidden_{shared}\right)\right),\;hidden_{DO}=\;\mathrm{ReLU}\left({\mathrm{MLP}}_{hidden}\left(out_{DO},hidden_{shared}\right)\right),\;hidden_{COD}=\mathrm{ReLU}\left({\mathrm{MLP}}_{hidden}\left(out_{COD},hidden_{shared}\right)\right),\;hidden_{NHN}=\mathrm{ReLU}({\mathrm{MLP}}_{hidden}\left(out_{NHN},hidden_{shared}\right)).$$

The vector input of the tower layer is obtained by weighted fusion:

(23) $$v_{pH}=W_{pH}\times hidden_{pH}+W_{hidden}\times hidden_{pH},\;v_{DO}=W_{DO}\times hidden_{DO}+W_{hidden}\times hidden_{DO},\;v_{COD}=W_{COD}\times hidden_{COD}+W_{hidden}\times hidden_{COD},\;v_{NHN}=W_{NHN}\times hidden_{NHN}+W_{hidden}\times hidden_{NHN}.$$

We then input $v_{DO}$, $v_{COD}$ and $v_{NHN}$ into the corresponding tower layers. For different prediction indicators, the tower layer structure and output are different. Taking MLP as an example, the formula is shown as Equation (16) in Section 2.3.2. Finally, the RMSE between the predicted and real values is calculated as the loss, and the model parameters are updated by the backpropagation method until the model converges.

#### 2.3.5. Summary of Four Water Quality Prediction Models

The structure of the proposed four water quality prediction models is summarized in Table 1. The input layer and output are not listed in the table due to their similarity and simplicity. For more details, please refer to Appendix A.

## 3. Experiment Setup

This section introduces the datasets, evaluation metrics, baseline models, and model settings for the evaluation.

### 3.1. Datasets

The experiment datasets come from 147 water quality monitoring stations set up by China National Environmental Monitoring Station in China’s seven river systems and lakes. Each station’s monitoring water quality indicators include pH, DO, COD_Mn_, and NH_3_-N. We have two datasets: D-s (Dataset-short) from 2013 to 2015 and D-l (Dataset-long) from 2012 to 2018. We select 120 stations with relatively complete data as the experiment dataset. Among them, there are 7 monitoring stations in the Pearl River, 22 in the Yangtze River, 11 in the Songhua River, 7 in the Liaohe River, 12 in the Yellow River, 26 in the Huaihe River, 6 in the Haihe River, 6 in the Taihu Lake, 4 in Poyang Lake, and 18 in other large lakes and rivers. Detailed statistics of the dataset are shown in Table 2.

### 3.2. Evaluation Metrics

We select Root Mean Square Error (RMSE), Mean Absolute Percentage Error (MAPE), and Mean Absolute Error (MAE) as the evaluation metrics, which are widely used in time series prediction models [29]. Note that the lower the values of RMSE, MAPE, and MAE, the better the performance. The RMSE, MAE, and MAPE are calculated as follows:

(24) $$\mathrm{RMSE}=\sqrt{\frac{1}{N}\overset{N}{\underset{i=1}{\sum }}{\left(y_{i}-{\hat{y}}_{i}\right)}^{2}}$$

(25) $$\mathrm{MAE}=\frac{1}{N}\overset{N}{\underset{i=1}{\sum }}\left|y_{i}-{\hat{y}}_{i}\right|$$

(26) $$\mathrm{MAPE}=\frac{100\%}{N}\overset{N}{\underset{i=1}{\sum }}\left|\frac{y_{i}-{\hat{y}}_{i}}{y_{i}}\right|$$

where $y_{i}$ indicates the *i*-th real value, ${\hat{y}}_{i}$ indicates the *i*-th predicted value, and N is the number of data samples. These three metrics are used to measure the error between the predicted values and the real values. MAPE reflects the relative error between the predicted values and the real values, while MAE is a simple superposition of the absolute error. Therefore, MAPE can more accurately reflect the deviation degree of the predicted values. At the same time, RMSE first squares the error values. If the dispersion of errors is high, the RMSE is magnified. Therefore, RMSE is more affected by outliers than MAE and MAPE, but they are at the same data level [30].

### 3.3. Baselines

The baselines for comparison are as follows: Linear model [16], XGBoost model [31,32], MLP model [33], CNN model [34], LSTM model [19], GRU (Gated Recurrent Unit) model [35], and ATTENTION model (ATT for short) [36,37]. Our proposed models are Mt-Hard (Multi-Task-Hard), Mt-Soft (Multi-Task-Soft), Mt-Gate (Multi-Task-Gate), and Mt-GH (Multi-Task-GH).

### 3.4. Model Setting

For model learning, the input space node number is 120, the sequence length is 10, and the dimension of each time point is 4, representing four water quality indicators (pH, DO, COD_Mn_, and NH_3_-N). For prediction, the output space node number is also 120, the sequence length is set to 1, and the water quality indicators at each time point are also pH, DO, COD_Mn_, and NH_3_-N. The first 60% of the data is used for training, 20% is used for validation, and the last 20% is used for testing. The prediction time step is set to 1. In other words, the historical water quality values of 120 monitoring stations in the previous ten weeks are used to predict their values in the next week. We compare the proposed models with other models to verify the effectiveness of the proposed models.

For all deep learning models, Adam is used as the optimizer, which combines the advantages of AdaGrad (adaptive gradient) and RMSProp (root mean square propagation) to update the step size by comprehensively considering the first-moment estimation (i.e., the mean value of the gradient) and the second-moment estimation (i.e., the variance of the gradient). The learning rate can be automatically adjusted, and the fluctuation range of the adjustment is not too large [29]. The hyperparameters are highly interpretable and usually only need to be fine-tuned or even not need to be adjusted, which is suitable for large-scale data and parameter scenarios. We choose RMSE as the loss function. The learning rate is set to 0.001, and the epochs and batch sizes are set to 100 and 5, respectively.

## 4. Results and Discussion

In this section, we compare the proposed method with baselines on the prediction performance of the single-indicator and multi-indicator. We then compare the influence of different tower layers on the model to verify the proposed methods’ robustness and analyze the models’ predictive performance for different rivers and lakes. We also show the training loss and validation loss of the best multi-task water quality prediction model.

### 4.1. Comparison of Prediction Performance for Single-Indicator

In this section, we compare the overall prediction performance of four multi-task learning models with seven baselines for single-indicator. The experimental results are shown in Table 3. In the table, the bold numbers are the best, and the numbers with asterisk are the second best.

(1) For pH, the hard parameter sharing structure (Multi-Task-Hard), soft parameter sharing structure (Multi-Task-Soft), gated parameter sharing structure (Multi-Task-Gate), and gated hidden parameter sharing structure (Multi-Task-GH) achieve better performance in all the metrics. Multi-Task-GH achieves the best performance, which means the pH predicted by Multi-Task-GH is closer to the real values.

(2) For DO, the four multi-task learning models also achieve better performance. Among the four multi-task learning models, the performance of Multi-Task-Hard is worse than other multi-task models (Multi-Task-Soft, Multi-Task-Gate, and Multi-Task-GH) and even worse than some traditional deep learning models (MLP and GRU). The Multi-Task-GH still achieves the best performance.

(3) For COD_Mn_, the MLP model achieves the best performance in RMSE and MAE. Only Multi-Task-Gate achieves the best performance in MAPE among the four multi-task learning models. The prediction performance of the three soft parameter sharing models, Multi-Task-Soft, Multi-Task-Gate, and Multi-Task-GH, is almost the same as that of MLP, which means that the predicted COD_Mn_ of MLP is closer to the observed values. However, the multi-task learning model with three soft parameters shared can still achieve close results.

(4) For NH_3_-N, MLP achieves the best performance in only MAPE, while Multi-Task-GH achieves the best results in both RMSE and MAE. This means that the NH_3_-N predicted by the Multi-Task-GH model is closer to the real values in most cases.

As shown in Table 4, we further validate the proposed models on the dataset D-l. In the table, the bold numbers are the best. Similar to the results on D-s, the results also show that the multi-task learning models achieve better performance than other models in most cases and Multi-Task-GH achieves the best results in most.

### 4.2. Comparison of Four Indicators and Three Indicators Multi-Task Learning Models

It is worth mentioning that we have conducted experiments on three indicators of multi-task learning models. The experimental results show a similar conclusion, but the whole performance is weaker than the four ones. The results are shown in Table 5, where 4-task means four indicator multi-task learning model, 3-tasks are three indicator multi-task learning models that include (pH, DO, COD_Mn_), and (DO, COD_Mn_, NH_3_-N), (pH, DO, NH_3_-N), and (pH, COD_Mn_, NH_3_-N) multi-task learning models. In the table, the bold numbers are the best. The results on D-s have similar trends.

### 4.3. Comparison of Prediction Performance for Multi-Indicators

Table 6 shows the average prediction performance of seven baselines and four multi-task learning models for multi-indicators on D-s. In the table, the bold numbers are the best, and the numbers with asterisk are the second best. For the space limitation, we only put the results on D-s in the following sections because the results on D-s and D-l have similar trends. Among the models, the Multi-Task-GH model achieves the best on all the metrics. Although the single-task learning models may achieve the best effect in predicting one target water quality indicator in some cases, the prediction accuracy will decrease when predicting other water quality indicators. Therefore, when the same model structure is used to simultaneously predict multiple target water quality indicators (pH, DO, COD_Mn_, and NH_3_-N), the Multi-Task-GH model can accomplish this task well and achieve the best performance in most indicators. This means that the Multi-Task-GH model can accurately predict multi-indicator.

### 4.4. Tower Layer Analysis

This paper also analyzes the impact of different tower types on the prediction performance of the Multi-task-GH model, as shown in Table 7. In the table, the bold numbers are the best. The five deep learning structures of LSTM, GRU, CNN, ATTENTION, and MLP are used as the tower layer of the Multi-Task-GH model to train the model and predict the water quality indicators. The results show that the Multi-Task-GH model with MLP as the tower layer achieves the best performance in most cases. The robustness of the model is the best, and there is no sharp drop in the prediction accuracy when predicting different water quality indicators. For example, when the ATTENTION-based deep learning structure is used as the tower layer of the Multi-Task-GH model, the prediction results of DO, COD_Mn_, and NH_3_-N are good, while the prediction results of pH are greatly reduced, which achieve the worst performance among the five structures. This shows that the ATTENTION-based deep learning structure is not well compatible with the simultaneous prediction of four water quality indicators.

### 4.5. The Difference of Predictions and Real Data

**Figure 7:**
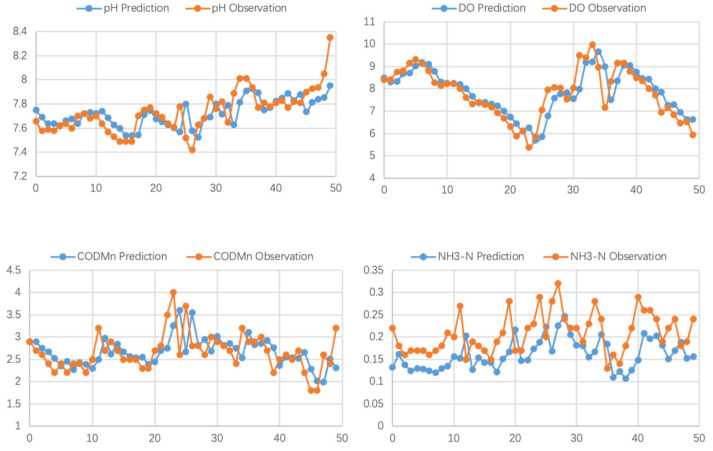
The difference between predictions and real data.

To show the ability of the Multi-Task-GH model, we the difference between predictions and the real measured data, as shown in Figure 7. The curves of both present similar trends, which prove the Multi-Task-GH model can predict the indicator change.

### 4.6. Model Training Loss and Validation Loss

**Figure 8:**
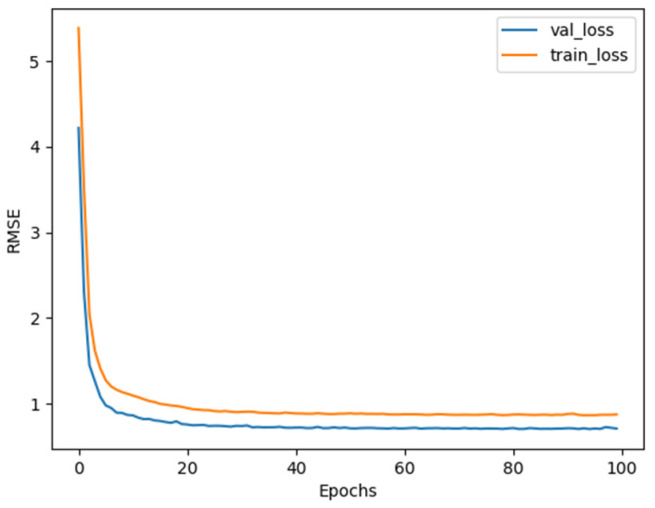
The learning curve of the Multi-Task-GH model.

We train the baselines and the proposed four multi-task learning models with fixed hyperparameters. As shown in Figure 8, the two curves are the training loss and validation loss change curves of the Multi-Task-GH model. The two-loss curves converge after about ten epochs of training.

### 4.7. Related Work Analysis

With the rapid development of machine learning, scholars have begun to explore water quality prediction methods based on machine learning, such as the support vector machine, genetic algorithm, and clustering algorithm. Recently, some researchers have proposed deep learning methods for water quality prediction, mainly aimed at predicting a single indicator. These models are based on single-task learning, and the representative models are as follows:

Avila et al. [16] adopted the ridge regression method to predict water quality. Lu et al. [31] used PCA to assess water quality. Chen et al. [32] used a machine learning algorithm with an integrated boosting method. Ahmed et al. [33] stacked multiple fully connected layers to predict water quality. Barzegar et al. [34] employed one convolution layer and LSTM layers for water quality parameter prediction. Yang et al. [21] incorporated one LSTM and two fully connected layers for prediction, which can extract short-term and long-term correlations of water quality and avoid gradient disappearance. Shrestha et al. [35] incorporated one GRU and two fully connected layers for water quality prediction.

Vaswani et al. and Jaderberg et al. [36,37] stacked three self-attention layers and two fully connected layers to mine the sequential relationship of water quality data. The input sequence is first converted to embedding through the first fully connected layer. The converted embedding then completes the information aggregation on the time step through the three-layer self-attention mechanism. Finally, it generates the water quality prediction through a fully connected layer.

Prediction methods based on deep learning can well extract the complex nonlinear characteristics and time-dependent relationship of water quality data, which achieves good prediction performance, but they still have some problems.

(1) Unable to predict multiple indicators with one model. The trained model often only performs well in the one prediction indicator. If the model is used to predict other indicators without changing the model’s structure and parameters, the performance will be greatly reduced. Therefore, it is necessary to train different models to predict multiple indicators, which will lead to extended training and prediction time and large model storage space.

(2) Unable to consider the impact correlations between multiple indicators. When the water quality of the same water area becomes better or worse, there may be a certain correlation between different indicators. The single-indicator prediction model is difficult to deal with the correlations between multiple indicators.

This paper proposes water quality prediction models based on multi-task learning to solve the above problems. The model based on multi-task learning improves the prediction performance of each task, which learns the relevance between different tasks. The multi-task learning also saves parameter space and prediction time consumption by sharing part of the model [38,39].

## 5. Conclusions

This paper proposed a multi-task-learning-based prediction method to solve the shortcomings and challenges of the single-task learning model for water quality prediction. Four multi-task learning structures are proposed based on the idea of sharing bottom structures: hard parameter sharing structure, soft parameter sharing structure, gated parameter sharing structure, and gated hidden parameters sharing structure. Sufficient experiments are designed and implemented to demonstrate the effectiveness of the proposed method.

However, there is still room for improving the proposed method. The training gradient losses of different tasks in reverse gradient propagations show a magnitude gap, leading to unstable training. It is hard to train a large number of water quality indicators in multi-task learning because the balance of four indicators will be out of control.

In this paper, we did not take the data distributions and importance of different tasks into consideration explicitly because the Multi-task-GH can implicitly learn the unique and shared joint weights of each subtask through the gate network, and it can also implicitly reflect the different effects of data distributions. However, if the data distributions and importance of different tasks can be explicitly taken into consideration, e.g., as constraints of loss function or regularization, the models would have better performance. In the future, we will conduct more data analysis and design more reasonable losses for the tasks.

## Figures and Tables

**Table 1:** The structure of the proposed four water quality prediction models.

Name	Layer	Design
Mt-Hard	Shared parameter layer	1 × (MLP + Relu)
	Tower layer	pH: 3 × (MLP + ReLU) DO: 3 × (MLP + ReLU) COD_Mn_: 2 × (MLP + ReLU) NH_3_-N: 2 × (MLP + ReLU)
Mt-Soft	Shared parameter layer	pH, DO, COD_Mn_, NH_3_-N: 1 × (MLP + ReLU) Hidden: 2 × (MLP + ReLU)
	Tower layer	pH: 3 × (MLP + ReLU) DO: 3 × (MLP + ReLU) COD_Mn_: 2 × (MLP + ReLU) NH_3_-N: 2 × (MLP + ReLU)
Mt-Gate	Shared parameter layer	pH, DO, COD_Mn_, NH_3_-N: 1 × (MLP + ReLU)
	Tower layer	pH: Softmax + 3 × (MLP + ReLU) DO: Softmax + 3 × (MLP + ReLU) COD_Mn_: Softmax + 2 × (MLP + ReLU) NH_3_-N: Softmax + 2 × (MLP + ReLU)
Mt-GH	Shared parameter layer	pH, DO, COD_Mn_, NH_3_-N: 1 × (MLP + ReLU) Hidden: 2 × (MLP + ReLU)
	Tower layer	pH: Softmax + 3 × (MLP + ReLU) DO: Softmax + 3 × (MLP + ReLU) COD_Mn_: Softmax + 2 × (MLP + ReLU) NH_3_-N: Softmax + 2 × (MLP + ReLU)

**Table 2:** Dataset statistics.

Name	Number of Sites	D-s	D-l
		Time	Time
Total data set	120	2013.1–2015.2	2012.6–2018.4
Pearl River	8	2013.1–2015.2	2012.6–2018.4
The Yangtze River	22	2013.1–2015.2	2012.6–2018.4
Songhua River	11	2013.1–2015.2	2012.6–2018.4
Liaohe River	7	2013.1–2015.2	2012.6–2018.4
The Yellow River	12	2013.1–2015.2	2012.6–2018.4
Huaihe River	26	2013.1–2015.2	2012.6–2018.4
Haihe River	6	2013.1–2015.2	2012.6–2018.4
Taihu Lake	6	2013.1–2015.2	2012.6–2018.4
Poyang Lake	4	2013.1–2015.2	2012.6–2018.4
Other	18	2013.1–2015.2	2012.6–2018.4

**Table 3:** Comparison of the overall performance of prediction for single-indicator on D-s dataset.

Model	pH			DO			COD_Mn_			NH_3_-N		
	RMSE	MAE	MAPE	RMSE	MAE	MAPE	RMSE	MAE	MAPE	RMSE	MAE	MAPE
Linear [16]	0.560	0.413	0.054	2.657	1.978	0.299	2.793	1.236	0.354	1.366	0.423	0.948
XGB [31]	0.327	0.245	0.032	1.594	1.135	0.121	1.732	0.614	0.179	0.487	0.182	0.335
MLP [33]	0.299	0.211	0.028	1.218 *	0.828	0.910	**1.485**	**0.588**	0.178	0.474	0.164 *	**0.317**
CNN [34]	0.429	0.327	0.043	2.066	1.506	0.167	2.541	1.197	0.350	0.718	0.347	0.702
LSTM [19]	0.294	0.208	0.027	1.396	0.956	0.103	1.658	0.617	0.174	0.467	0.171	0.444
GRU [35]	0.282 *	0.206 *	0.027 *	1.230	0.821 *	0.087 *	1.742	0.610	0.169	0.457 *	0.172	0.434
ATT [37]	0.478	0.387	0.050	1.672	1.079	0.106	2.035	0.618	0.168 *	0.681	0.193	0.416
Mt-Hard	0.270	0.217	0.028	1.273	0.869	0.094	1.535	0.602	0.169	0.432	0.244	0.640
Mt-Soft	0.293	0.209	0.028	1.186	0.801	0.087	1.534	0.597	0.178	0.430	0.168	0.386
Mt-Gate	0.292	0.211	0.027	1.235	0.851	0.091	1.547	0.585	**0.166**	0.448	0.178	0.386
Mt-GH	**0.262**	**0.181**	**0.024**	**1.182**	**0.796**	**0.086**	1.515	0.592	0.173	**0.403**	**0.154**	0.331
Improv.	7.1%	12.1%	11.1%	3.0%	3.0%	1.1	-	-	1.6%	11.7%	6.3%	-

* In the table, the bold numbers are the best, and the numbers with asterisk are the second best.

**Table 4:** Comparison of the overall performance of prediction for single-indicator on D-l dataset.

Model	pH			DO			COD_Mn_			NH_3_-N		
	RMSE	MAPE	MAE	RMSE	MAPE	MAE	RMSE	MAPE	MAE	RMSE	MAPE	MAE
CNN	0.456	0.047	0.357	2.049	0.192	1.547	1.487	0.346	1.051	0.323	0.946	0.188
LSTM	0.268	0.025	0.195	1.198	0.110	0.828	0.939	0.189	0.582	0.238	0.430	0.118
GRU	**0.242**	**0.022**	**0.168**	1.200	0.107	**0.813**	0.944	0.190	0.579	0.219	0.510	0.116
Mt-Soft	0.252	0.023	0.178	1.196	0.106	0.823	0.949	0.188	0.577	0.222	0.395	0.114
Mt-Gate	0.251	0.023	0.178	1.197	0.108	0.823	0.939	0.194	0.582	0.217	0.415	0.109
Mt-GH	0.256	0.023	0.181	**1.196**	**0.106**	0.824	**0.932**	**0.185**	**0.574**	**0.214**	**0.407**	**0.105**
Mt-GH	0.256	0.023	0.181	**1.196**	**0.106**	0.824	**0.932**	**0.185**	**0.574**	**0.214**	**0.407**	**0.105**

**Table 5:** Comparison of four indicators and three indicators multi-task learning models on D-l.

	pH			DO			COD_Mn_			NH_3_-N		
	RMSE	MAPE	MAE	RMSE	MAPE	MAE	RMSE	MAPE	MAE	RMSE	MAPE	MAE
4-task	**0.256**	**0.023**	**0.181**	**1.196**	**0.106**	**0.824**	**0.932**	**0.185**	**0.574**	**0.214**	**0.407**	**0.105**
3-task1	0.324	0.031	0.235	1.248	0.116	0.877	0.959	0.194	0.592	—	—	—
3-task2	—	—	—	1.277	0.116	0.892	0.965	0.193	0.594	0.234	0.514	0.136
3-task3	0.316	0.030	0.226	1.242	0.111	0.861	—	—	—	0.222	0.650	0.127
3-task4	0.342	0.033	0.255	—	—	—	0.977	0.189	0.596	0.221	0.440	0.107

**Table 6:** Comparison of the overall performance of prediction for multi-indicator.

Model	RMSE	MAE	MAPE
Linear [16]	7.376	4.052	1.656
XGB [31]	4.139	2.177	0.667
MLP [33]	3.476 *	1.792 *	0.615 *
CNN [34]	5.753	3.377	1.262
LSTM [19]	3.814	1.952	0.748
GRU [35]	3.709	1.809	0.716
ATT [37]	4.866	2.277	0.730
Mt-Hard	3.511	1.932	0.930
Mt-Soft	3.443	1.775	0.679
Mt-Gate	3.523	1.823	0.670
Mt-GH	**3.362**	**1.723**	**0.614**
Improv.	3.3%	3.8%	1.6%

* In the table, the bold numbers are the best, and the numbers with asterisk are the second best.

**Table 7:** The performance comparison of tower structures in the Multi-Task-GH model.

Tower Type	pH			DO			COD_Mn_			NH_3_-N		
	RMSE	MAE	MAPE	RMSE	MAE	MAPE	RMSE	MAPE	MAE	RMSE	MAPE	MAE
LSTM	7.009	6.975	0.904	9.178	8.872	0.919	4.523	0.735	2.978	1.080	0.515	0.343
GRU	0.847	0.396	0.051	2.311	1.642	0.172	2.705	0.369	1.313	1.120	0.627	0.423
CNN	0.464	0.359	0.046	1.949	1.407	0.154	2.576	0.371	1.304	0.950	0.896	0.367
ATT	7.725	7.708	1.0	1.459	0.952	0.098	1.987	**0.162**	0.614	0.474	0.430	0.186
MLP	**0.262**	**0.181**	**0.024**	**1.182**	**0.796**	**0.086**	**1.515**	0.173	**0.592**	**0.403**	**0.331**	**0.154**

## Data Availability

Publicly available datasets were analyzed in this study. This data can be found here: http://envi.ckcest.cn/environment/special/special_list.jsp?specialId=108, accessed on 10 October 2021.

## Appendix A. Hyper-Parameters

In our proposed framework, there are many hyper-parameters. For MLP, we tune the number of hidden layers in a range of {1, 2, 3} and tune the number of hidden units in a range of {16, 32, 64}. Without specific mention, we set hidden units of the first MLP layer as 64, the second MLP layer as 32, and the third MLP layer as 16. We further tune the number of epochs for the training process in a range of {100, 200, 400}, the number of batch size is 8, and the learning rate is 0.001. The input space node number is 120, the sequence length is 10, and the dimension of each time point is 4, representing four water quality indicators: pH, DO, COD_Mn_, and NH_3_-N. All the information is summarized in Table A1.

**Table A1:** Hyper-parameters.

Name	Experimental Set
MLP layer	1, 2, 3 6.97 0.904
MLP hidden unit units	16, 32, 64 0.396 0.051
Epoch	100, 200, 400 0.359 0.046
Batch size	8 7.708 1.0
Learning rate	0.001 0.181 0.024
node number	120
Sequence length	10
Prediction targets	4

Table A2 shows the details of input-output parameters.

**Table A2:** The details of input-output parameters.

Inputs	Outputs	Time Windows Size	Mean	Standard Variance	Maximum	Minimum
pH	pH	10	7.240	0.330	7.950	6.390
DO	DO	10	7.340	0.640	10.000	6.200
COD_Mn_	COD_Mn_	10	1.820	0.450	3.300	0.800
NH_3_-N	NH_3_-N	10	0.194	0.045	0.340	0.110

## References

[1] Votruba L. (1988). Analysis of Water Resource Systems.

[2] Olyaie E., Abyaneh H.Z., Mehr A.D. (2017). A comparative analysis among computational intelligence techniques for dissolved oxygen prediction in Delaware River. Geosci. Front..

[3] Cortes C., Vapnik V. (1995). Support-vector networks. Mach. Learn..

[4] Drucker H. Improving regressors using boosting techniques. Proceedings of the 14th International Conference on Machine Learning.

[5] Vapnik V., Golowich S., Smola A., Mozer M.C., Jordan M., Petsche T. (1997). Support Vector Method for Function Approximation, Regression Estimation and Signal Processing. Advances in Neural Information Processing Systems.

[6] Li X., Cheng Z., Yu Q., Bai Y., Li C. (2017). Water-quality prediction using multimodal support vector regression: Case study of Jialing River, China. J. Environ. Eng..

[7] Huang N.E., Shen Z., Long S.R. (1998). The empirical mode decomposition and the Hilbert spectrum for nonlinear and nonstationary time series analysis. Process R. Soc. Lond..

[8] Wu Z., Huang N.E. (2009). Ensemble empirical mode decomposition: A noiseassisted data analysis method. Adv. Adapt. Data Anal..

[9] Yeh J.R., Shieh J.S., Huang N.E. (2010). Complementary ensemble empirical mode decomposition: A novel noise enhanced data analysis method. Adv. Adapt. Data Anal..

[10] Leong W.C., Bahadori A., Zhang J., Ahmad Z. (2021). Prediction of water quality index (WQI) using support vector machine (SVM) and least square-support vector machine (LS-SVM). Int. J. River Basin Manag..

[11] Rashed E.A., Hirata A. (2021). Infectivity upsurge by COVID-19 viral variants in Japan: Evidence from Deep Learning Modeling. Int. J. Environ. Res. Public Health.

[12] Dildar M., Akram S., Irfan M., Khan H.U., Ramzan M., Mahmood A.R., Alsaiari S.A., Saeed A.H.M., Alraddadi M.O., Mahnashi M.H. (2021). Skin cancer detection: A review using deep learning techniques. Int. J. Environ. Res. Public Health.

[13] Banejad H., Olyaie E. (2011). Application of an artificial neural network model to rivers water quality indexes prediction—A case study. J. Am. Sci..

[14] Heddam S. (2016). Multilayer perceptron neural network-based approach for modeling pHycocyanin pigment concentrations: Case study from lower Charles River buoy, USA. Environ. Sci. Pollut. Res..

[15] Heddam S. (2014). Generalized regression neural network-based approach for modeling hourly dissolved oxygen concentration in the Upper Klamath River, Oregon, USA. Environ. Technol..

[16] Avila R., Horn B., Moriarty E., Hodson R., Moltchanova E. (2018). Evaluating statistical model performance in water quality prediction. J. Environ. Manag..

[17] Zhou Z.H., Feng J. (2019). Deep Forest. Natl. Sci. Rev..

[18] Wang Z., Man Y., Hu Y., Li J., Hong M., Cui P. (2019). A deep learning based dynamic COD prediction model for urban sewage. Environ. Sci. Water Res. Technol..

[19] Zou Q., Xiong Q., Li Q., Yi H., Yu Y., Wu C. (2020). A water quality prediction method based on the multi-time scale bidirectional long short-term memory network. Environ. Sci. Pollut. Res..

[20] Niu C., Tan K., Jia X., Wang X. (2021). Deep learning based regression for optically inactive inland water quality parameter estimation using airborne hyperspectral imagery. Environ. Pollut..

[21] Yang Y., Xiong Q., Wu C., Zou Q., Yu Y., Yi H., Gao M. (2021). A study on water quality prediction by a hybrid CNN-LSTM model with attention mechanism. Environ. Sci. Pollut. Res..

[22] Guo H., Tian S., Huang J.J., Zhu X., Wang B., Zhang Z. (2022). Performance of deep learning in mapping water quality of Lake Simcoe with long-term Landsat archive. ISPRS J. Photogramm. Remote Sens..

[23] Chen K., Chen H., Zhou C., Huang Y., Qi X., Shen R., Liu F., Zuo M., Zou X., Wang J. (2020). Comparative analysis of surface water quality prediction performance and identification of key water parameters using different machine learning models based on big data. Water Res..

[24] Zhong F., Wu J., Dai Y., Deng Z., Cheng S. (2019). Responses of water quality and phytoplankton assemblages to remediation projects in two hypereutrophic tributaries of Chaohu Lake. J. Environ. Manag..

[25] Weinberger K., Dasgupta A., Langford J., Smola A., Attenberg J. Feature hashing for large scale multi-task learning. Proceedings of the 26th Annual International Conference on Machine Learning.

[26] Huang W., Song G., Hong H., Xie K. (2014). Deep architecture for traffic flow prediction: Deep belief networks with multi-task learning. IEEE Trans. Intell. Transp. Syst..

[27] Mao C., Gupta A., Nitin V., Ray B., Song S., Yang J., Vondrick C. (2020). Multi-task learning strengthens adversarial robustness. European Conference on Computer Vision.

[28] Yu F., Chen H., Wang X., Xian W., Chen Y., Liu F., Madhavan V., Darrell T. Bdd100k: A diverse driving dataset for heterogeneous multi-task learning. Proceedings of the IEEE/CVF Conference on Computer Vision and Pattern Recognition (CVPR).

[29] Willmott C.J., Matsuura K. (2005). Advantages of the mean absolute error (MAE) over the root mean square error (RMSE) in assessing average model performance. Clim. Res..

[30] Kingma D.P., Ba J. (2014). Adam: A method for stochastic optimization. arXiv.

[31] Lu W., Wu J., Li Z., Cui N., Cheng S. (2019). Water quality assessment of an urban river receiving tail water using the single-factor index and principal component analysis. Water Sci. Tech..

[32] Chen T., Guestrin C. Xgboost: A scalable tree boosting system. Proceedings of the 22nd ACM SIGKDD International Conference on Knowledge Discovery and Data Mining.

[33] Ahmed AA M. (2017). Prediction of dissolved oxygen in Surma River by biochemical oxygen demand and chemical oxygen demand using the artificial neural networks (ANNs). J. King Saud Univ.-Eng. Sci..

[34] Barzegar R., Aalami M.T., Adamowski J. (2020). Short-term water quality variable prediction using a hybrid CNN–LSTM deep learning model. Stoch. Environ. Res. Risk Assess..

[35] Jiang Y., Li C., Sun L., Guo D., Zhang Y., Wang W. (2021). A deep learning algorithm for multi-source data fusion to predict water quality of urban sewer networks. J. Clean. Prod..

[36] Vaswani A., Shazeer N., Parmar N., Uszkoreit J., Jones L., Gomez A.N., Kaiser Ł., Polosukhin I. (2017). Attention is all you need. Adv. Neural Inf. Processing Syst..

[37] Liu Y., Zhang Q., Song L., Chen Y. (2019). Attention-based recurrent neural networks for accurate short-term and long-term dissolved oxygen prediction. Comput. Electron. Agric..

[38] Collobert R., Weston J. A unified architecture for natural language processing: Deep neural networks with multi-task learning. Proceedings of the 25th International Conference on Machine Learning.

[39] Lindbeck A., Snower D.J. (2000). Multitask learning and the reorganization of work: From Taylorism to holistic organization. J. Labor Econ..

